# Unimpaired Skin Carcinogenesis in *Desmoglein 3* Knockout Mice

**DOI:** 10.1371/journal.pone.0050024

**Published:** 2012-11-21

**Authors:** Sylvain Baron, Anabel Hoang, Hannes Vogel, Laura D. Attardi

**Affiliations:** 1 Division of Radiation and Cancer Biology, Department of Radiation Oncology, Stanford University School of Medicine, Stanford, California, United States of America; 2 Department of Pathology, Stanford University School of Medicine, Stanford, California, United States of America; 3 Department of Genetics, Stanford University School of Medicine, Stanford, California, United States of America; Ohio State University Medical Center, United States of America

## Abstract

The contribution of adherens junction inactivation, typically by downregulation or mutation of the transmembrane core component E-cadherin, to cancer progression is well recognized. In contrast, the role of the desmosomal cadherin components of the related cell-cell adhesion junction, the desmosome, in cancer development has not been well explored. Here, we use mouse models to probe the functional role of desmosomal cadherins in carcinogenesis. Because mice lacking the desmosomal cadherin Desmoglein 3 (Dsg3) have revealed a crucial role for Dsg3 in cell-cell adhesion in stratified epithelia, we investigate the consequence of Dsg3 loss in two models of skin carcinogenesis. First, using *Dsg3−/−* keratinocytes, we show that these cells display adhesion defects *in vitro* and compromised tumor growth in allograft assays, suggesting that Dsg3 enables tumor formation in certain settings. In contrast, using an autochthonous model for SCC development in response to chronic UVB treatment, we discover a surprising lack of enhanced tumorigenesis in *Dsg3−/−* mice relative to controls, unlike mice lacking the desmosomal component Perp. Accordingly, there is no defect in the apoptotic response to UVB or enhanced immune cell infiltration upon Dsg3 loss that could promote tumorigenesis. Thus, Dsg3 does not display a clear function as a tumor suppressor in these mouse skin cancer models. Continued unraveling of the roles of Dsg3 and other desmosomal constituents in carcinogenesis in different contexts will be important for ultimately improving cancer diagnosis, prognostication, and treatment.

## Introduction

The vast majority of human cancers, known as carcinomas, arise from epithelia. Defining the factors that govern the normal architecture and function of epithelia, and how these can be perturbed, is therefore essential for understanding cancer development. Critical for the integrity of epithelia are various intercellular adhesion junctions, including adherens junctions and desmosomes [1]. Whereas adherens junctions are fundamental both for intercellular adhesion in epithelia and for enabling the dynamic rearrangements of epithelia [2], desmosomes are pivotal for reinforcing adhesion between epithelial cells via anchorage to the intermediate filament network [1], [3]. Adherens junctions mediate cell-cell interaction through the extracellular domains of transmembrane classical cadherins, the prototype of which is E-cadherin, and then communicate with the actin cytoskeleton through interactions with beta-catenin and alpha-catenin [4], [5], [6], [7]. Similarly, desmosomes establish cell-cell contact through transmembrane desmosomal cadherins, the desmogleins (DSG1-4) and the desmocollins (DSC1-3), which associate by homophilic and heterophilic interactions [8], [9]. The cytoplasmic domains of these transmembrane cadherins interact with members of the armadillo protein family known as plakophilins (PKP1-3) and plakoglobin (PG), which then connect to the intermediate filament network through desmoplakins (DSP1 and 2). Another important desmosome component, revealed through genetic knockout and immunogold electron microscopy studies, is the tetraspan membrane protein Perp. *Perp* null mice display profound blistering of the oral mucosa and skin due to fewer and structurally abnormal desmosomes [10].

It has been well established that the adherens junction component E-cadherin has a central role in cancer. E-cadherin is mutated or downregulated during the progression of many human cancers to an invasive and metastatic stage [11], [12], [13], [14], [15], [16]. The importance of E-cadherin loss in cancer progression has been clearly demonstrated in mouse models *in vivo*, where the artificial maintenance of E-cadherin expression in a pancreas islet cell tumor model caused tumors to arrest at the adenoma stage, while inactivation of E-cadherin caused tumors to progress from adenomas to carcinoma [17]. Moreover, ablation of E-cadherin in a mouse model of mammary cancer promoted tumor initiation and metastasis [18]. Thus, E-cadherin is critical for preventing tumor progression.

**Figure 1:**
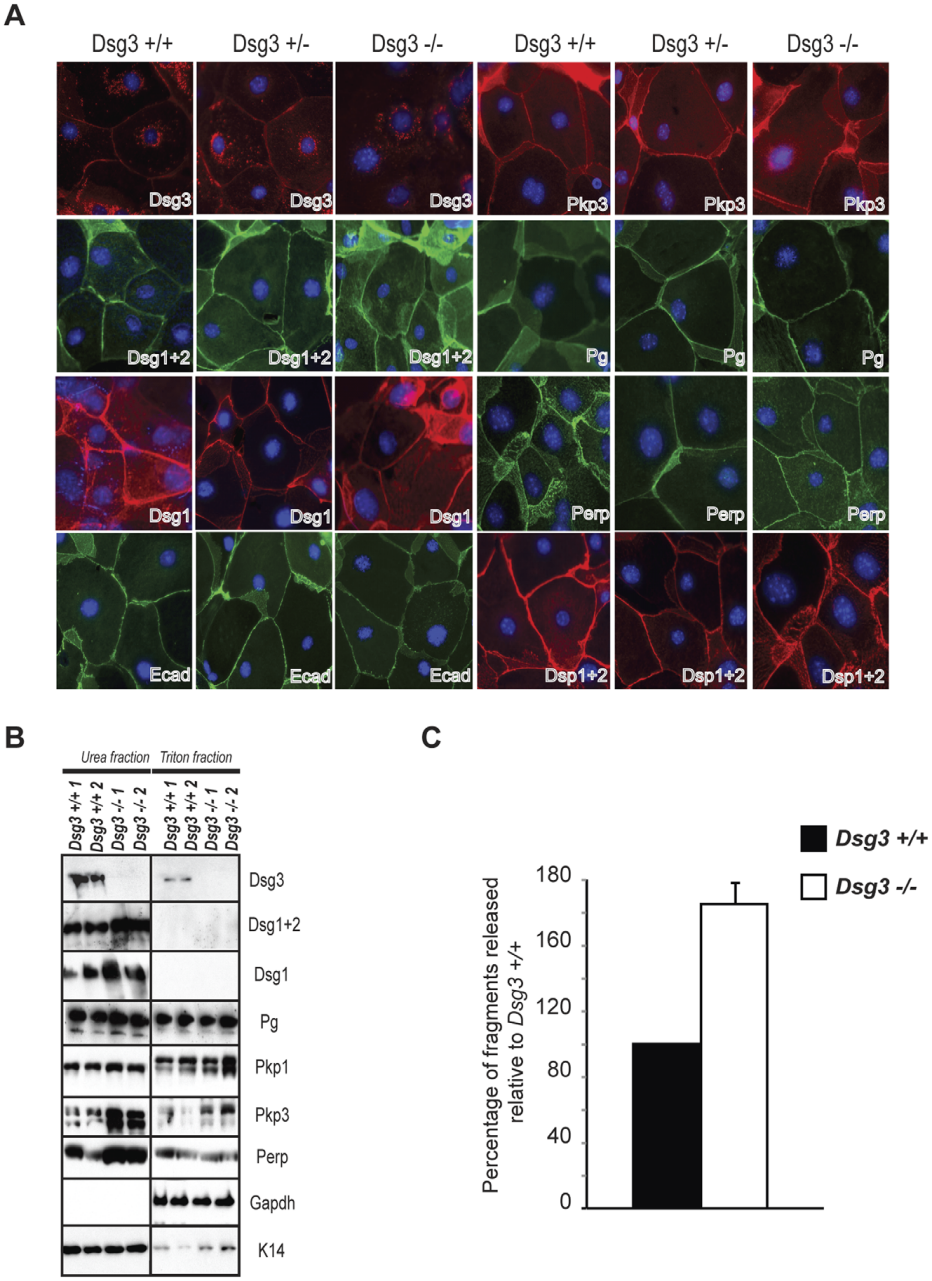
Dsg3 deficiency in keratinocytes does not dramatically affect localization or solubility of other desmosomal components, but does compromise cell-cell adhesion. A) Immunofluorescence analysis to examine localization of various desmosomal components in *Dsg3+/+, Dsg3+/−, Dsg3−/−* mouse keratinocyte monolayers after 48 hours of treatment with 2 mM Ca^2+^. DAPI is used as a nuclear marker (abbreviations: Dsg3 =  Desmoglein 3, Dsg1+2 =  Desmoglein 1 and 2, Dsg1 =  Desmoglein1, Ecad =  E-cadherin, Pkp3 =  Plakophilin 3, Pg =  Plakoglobin, Dsp1+2 =  Desmoplakin 1 and 2). B) Western blot analysis showing both the Triton X-100-soluble and urea-only soluble fractions of *Dsg3+/+* and *Dsg3−/−* mouse keratinocyte monolayers after 48 hours of treatment with 2 mM Ca^2+^. 1 and 2 denote two different keratinocyte samples. Gapdh serves as a loading control for the Triton X-100-soluble pool, while Keratin 14 serves as a loading control for the urea fraction. C) Graph indicating the average percentage (+/− SD) of fragments released from *Dsg3−/−* keratinocyte monolayers after mechanical stress relative to wild-type controls in a mechanical dissociation assay. Experiments were performed in triplicate. p<0.0001, Student’s t-test.

In contrast, the role of the desmosomal cadherins in carcinogenesis has remained unclear, in part because of contradictory reports in the literature. For example, loss of DSG1 expression has been observed during human skin carcinogenesis and is associated with worse prognosis in head and neck squamous cell carcinomas (SCCs) [19], [20], and loss of DSG3 and DSC3 expression was noted during human oral squamous carcinoma development, suggesting roles for these desmogleins as tumor suppressors [21]. In support of this notion, epigenetic silencing of *DSC3* is common in human breast cancer [22]. In contrast, DSG2 expression is increased in some malignant skin carcinomas [23], and transgenic expression of *Dsg2* in the differentiating layers of epidermis rendered mice susceptible to papilloma development, suggesting a pro-tumorigenic role for this desmosomal constituent [24]. Moreover, overexpression of DSG3 is correlated with malignant progression of human sinonasal inverted papillomas [25] and with development of cancers of the head, neck and lung [26], [27], also reflecting an oncogenic function for DSG3. An oncogenic role for DSG3 is further supported by *in vitro* experiments showing that DSG3 knockdown inhibits tumor growth and invasion in various head and neck cancer cell lines [26] and impairs epithelial cell proliferation [28]. These conflicting findings underscore the necessity for performing studies in mouse genetic models, in which the effects of modulating desmosomal constituents on carcinogenesis can be clearly interrogated through the use of knockout mice, to define the role of desmosomal cadherins in cancer development.

To better understand the role of desmosomal cadherins in cancer, we employed mice lacking the desmosomal cadherin Dsg3, which are viable yet in which Dsg3 plays key physiological roles in cell-cell adhesion, as *Dsg3* deficiency induces blisters of the oral mucosa and hair follicle defects. Here, we analyze the consequence of *Dsg3* nullizygosity for skin cancer development using two different tumor models. We first analyzed tumor development in an allograft model in which transformed keratinocytes with differing Dsg3 status were implanted subcutaneously into immunocompromised mice. We also used an autochthonous model to compare SCC incidence in *Dsg3−/−* mice relative to controls in response to chronic UVB treatment. Our analysis of the contribution of Dsg3 to cancer development in these two settings suggest that, unlike E-cadherin, Dsg3 loss is not sufficient to promote carcinogenesis and that Dsg3 does not display clear tumor suppressor activity.

**Figure 2:**
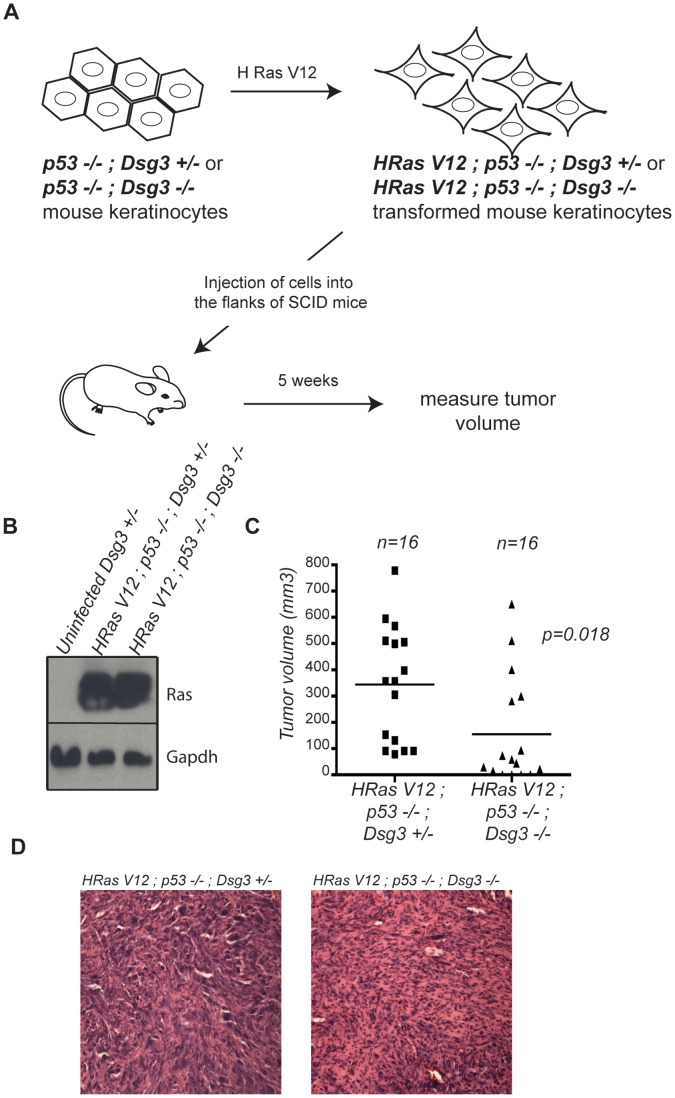
Dsg3 facilitates transformed keratinocyte allograft tumor growth. A) Experimental design for allograft tumor assays. B) Western blot analysis of *p53−/−;Dsg3+/−* and *p53−/−;Dsg3−/−* mouse keratinocytes transduced with HRasV12 lentiviruses confirms efficient HRasV12 expression relative to uninfected *Dsg3+/−* mouse keratinocytes. C) Graph displaying the volume of each tumor formed five weeks after implantation of *HRasV12;p53−/−;Dsg3+/−* (n = 16) and *HRasV12*;*p53−/−;Dsg3−/−* (n = 16) keratinocytes. p = 0.018, Student’s t-test. D) Representative hematoxylin and eosin (H and E)-stained sections of *HRasV12;p53−/−;Dsg3+/−* and *HRasV12;p53−/−;Dsg3−/−* tumors.

## Materials and Methods

### Keratinocyte Culture

Keratinocytes were derived from P0.5–P1.5 mouse skin as described [10]. Cells were grown on collagen/fibronectin-coated dishes and maintained in an undifferentiated state by growing the cells in low calcium EMEM (Lonza) containing 0.05 mM calcium, 8% dialyzed FCS, and antibiotics. Cells were then differentiated for 24 hrs in the same media as the undifferentiated cells, except that the calcium concentration was raised to 2 mM.

### Immunofluorescence

Immunofluorescence was performed essentially as described [29]. Primary antibodies used for immunofluorescence include the following: rabbit anti-Perp [10] (1∶200), mouse anti-desmoplakin clone 115F (gift from David Garrod, University of Manchester; 1∶50), chicken anti-plakoglobin 1408 (gift from Kathleen Green, Northwestern University; 1∶100), mouse anti-desmoglein 1+2 4B2 (gift from Kathleen Green, Northwestern University; 1∶100), goat anti-desmoglein 3 M-20 (Santa Cruz Biotechnology; 1∶50), mouse anti-desmoglein 1 18-D4 (Santa Cruz Biotechnology; 1∶100), mouse anti-plakophilin 3 23E3/4 (Zymed; 1∶250), rat anti-E-cadherin ECC-2 (Invitrogen; 1/500). Secondary antibodies used included FITC goat anti-rabbit (Vector Laboratories, 1∶300) and Alexa 546 donkey anti-mouse (Invitrogen; 1∶300). Fluorescence images were examined using a Leica DM6000B microscope (Leica Microsystems), and images were acquired using a Retiga Exi Camera (Q imaging) and Image Pro 6.2 software from Media Cybernetics.

**Figure 3:**
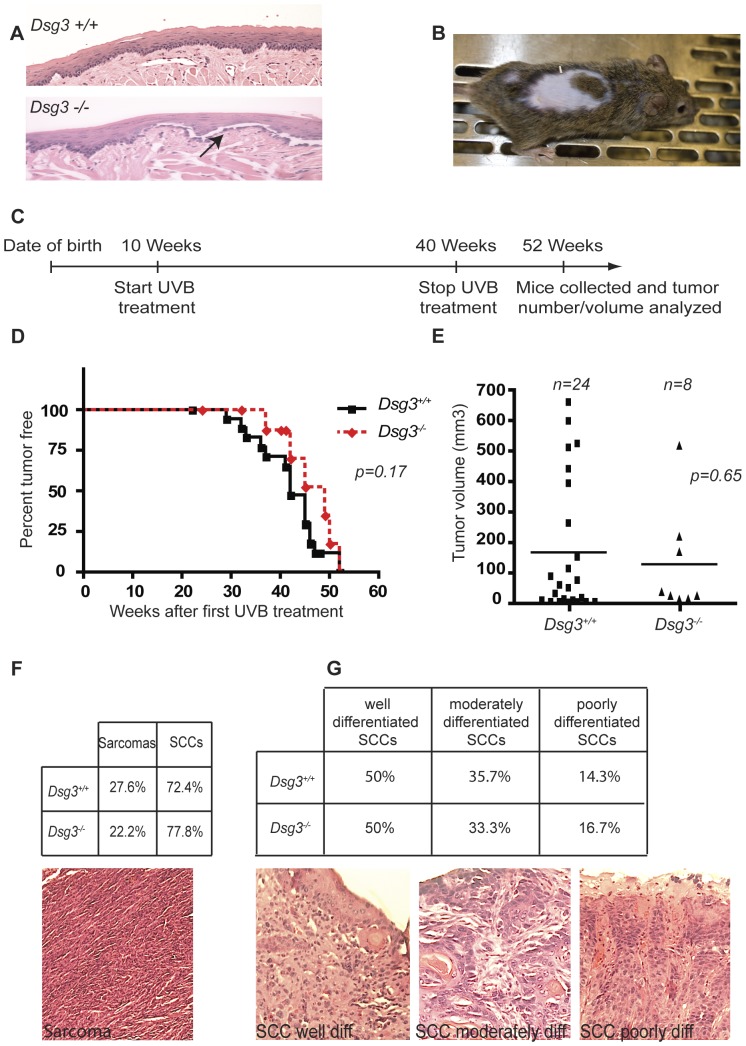
Dsg3 does not affect tumor incidence, tumor volume, or tumor grade in UVB-induced skin carcinogenesis. A) Representative hematoxylin and eosin (H and E)-stained sections of *Dsg3+/+* and *Dsg3−/−* mouse tongue epithelium. Arrow indicates the presence of blisters in the *Dsg3−/−* tongue epithelium. B) Photograph of an adult *Dsg3−/−* mouse, showing hair loss typical of *Dsg3* deficiency. C) Schematic diagram illustrating the experimental design and timeline for the UVB-induced SCC model used. D) Kaplan-Meier analysis of tumor-free survival of *Dsg3+/+* and *Dsg3−/−* mice subjected to chronic UVB treatment. p = 0.17, log rank test. E) Graph indicating the tumor volume in each *Dsg3+/+* (24 tumors collected) and *Dsg3−/−* (8 tumors collected) mouse after 52 weeks of UVB treatment. p = 0.65, Student’s t-test. F) Histological analysis by H and E staining, showing the percentages of sarcomas and squamous cell carcinomas among the tumors observed in mice of each genotype. A representative H and E-stained sarcoma is shown. G) Analysis of the percentages of SCCs of different grades in *Dsg3−/−* and *Dsg3+/+* mice. Photographs show representative H and E-stained SCCs with different levels of differentiation (highly, moderately, and poorly differentiated).

### Protein Preparation and Immunoblotting

For skin protein preparation, skin was snap-frozen and homogenized using a chilled mortar and pestle. For keratinocyte protein extracts, plated cells were washed with PBS and chilled buffer was added. For Triton-soluble protein fractions, cells or skin samples were resuspended in 1% Triton X-100/0.1% SDS solubilization buffer (150 mM NaCl, 20 mM Tris pH8, 1 mM EDTA, 0.5% NP40), rocked for one hour at 4°C, and the supernatant was isolated by centrifugation. The urea-soluble protein fraction was obtained by resuspending the Triton-insoluble material from this preparation in the same buffer +9M urea. Total protein extracts were made by direct lysis in 9M urea buffer. Western blotting was performed according to standard methods, with 25–50 µg of protein in each lane.

### Mechanical Dissociation Assay

The mechanical dissociation assay was performed as described previously [30]. Briefly, primary mouse epidermal keratinocytes were seeded in 6-well plates and grown to confluence in EMEM media containing 0.07 mM CaCl_2_. Cells were then differentiated for 24 hours by raising the calcium concentration in the media to 0.5 mM. After two washes with PBS, the adherent keratinocytes were incubated at 37°C for 30 minutes with 2.4 units of dispase I (Roche Applied Sciences), resulting in a non-adherent cell monolayer. The monolayers were carefully washed twice with PBS, transferred to 15 ml conical tubes and subjected to mechanical stress by inverting the conical tube thirty times. Cellular fragments were transferred to 35-mm tissue culture dishes and were imaged using a digital camera then counted. We performed these experiments in triplicate on three independent batches of mixed background *Dsg3+/+* and *Dsg3−/−* keratinocytes. The dispase assay was performed on each well, and 6 different fields were counted for each well, with the number of fragments per field ranging from 20 to 100. The number of fragments counted in each field (18 counts per genotype per experiment) was averaged and the standard deviation calculated. Statistical analysis was performed using the Student’s t-test.

**Figure 4:**
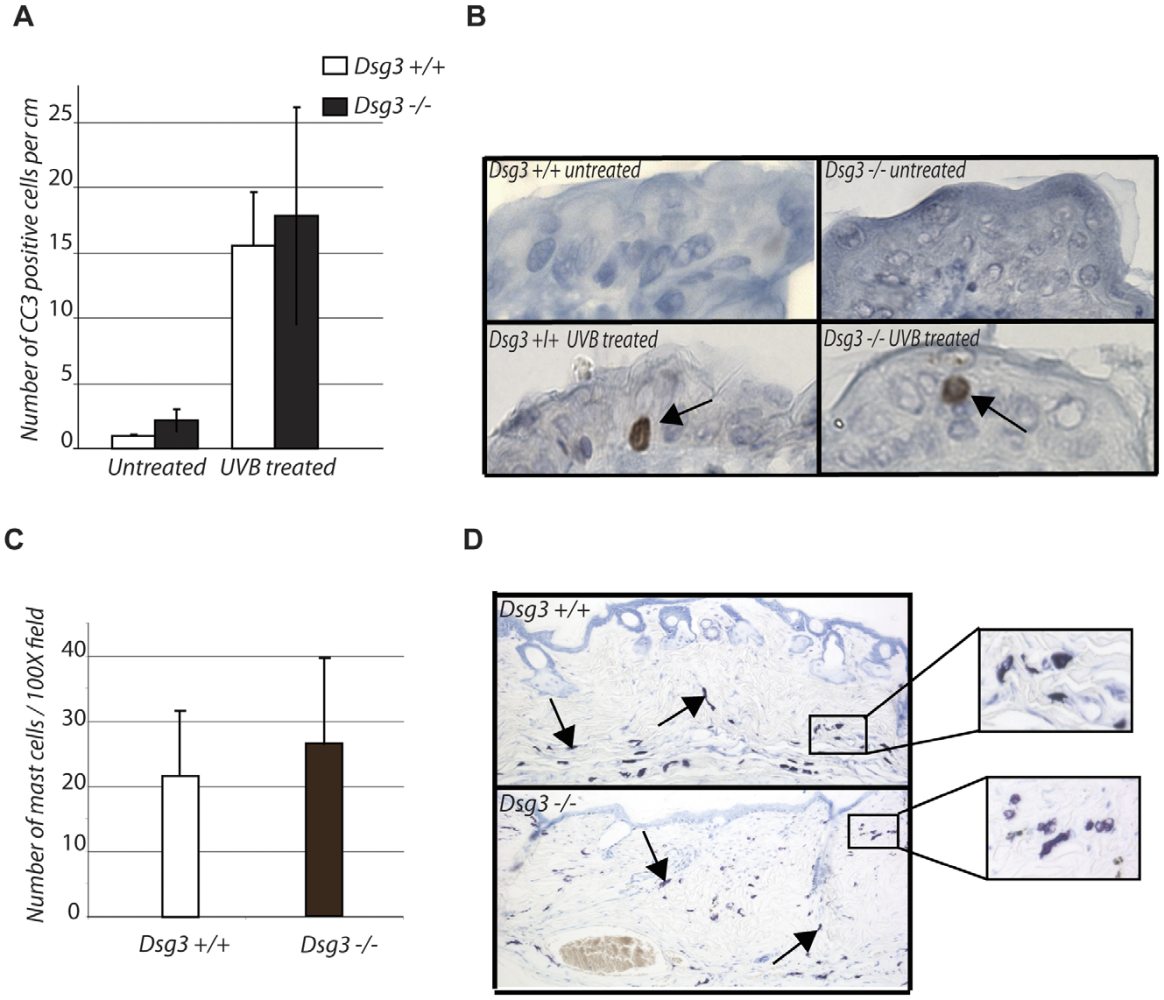
Dsg3 deficiency does not affect UVB-induced apoptosis or immune cell infiltration *in vivo.* A) Graph indicating the average number of cleaved Caspase 3 (CC3) positive cells +/− SD per cm of epidermis in *Dsg3+/+* and *Dsg3−/−* mice 24 hours after 2.5 kJ/m^2^ UVB. n = 3 for each condition. B) Representative images of cleaved Caspase 3 immunohistochemistry in the epidermis of mice analyzed in A). Arrows indicate the apoptotic cells. C) Graph indicating the average number of mast cells +/− SD in the skin of *Dsg3+/+* and *Dsg3−/−* mice after 52 weeks of UVB treatment. n = 12 for *Dsg3+/+* mice and n = 5 for *Dsg3−/−* mice. D) Representative toluidine blue staining of the skin of *Dsg3+/+* and *Dsg3−/−* mice after 52 weeks of UVB treatment. Arrows indicate mast cells stained by toluidine blue. Insets show higher magnification images of toluidine blue-stained skins, highlighting mast cells.

### Lentiviral Infection and Allograft Tumor Assays

Lentiviruses were produced by transiently transfecting 293 FT cells with 2.5 µg of packaging vectors (pMD2.G and psPAX2) along with 5 µg of a lentiviral vector encoding mutant H-RasV12 (gift from Michelle Marques, Stanford University). Infections were performed by incubating mouse keratinocytes with the produced virus in the presence of 8 µM polybrene for one hour. To increase the infection efficiency, cells were spun at 180 g during the process then subjected to selection for 72 hours in 5 µg/ml blasticidin. For the allograft tumor assays, 5×10^5^ transformed keratinocytes were injected into the flanks of *IcrTac:ICR-Prkdc^scid^* mice (Taconic). The mice were injected at 6 weeks of age, and tumors were allowed to grow for 5 weeks. Mice were then sacrificed, and tumor volume was measured with calipers.

### Ethics Statement

All animal studies were approved by the Stanford University Administrative Panel on Laboratory Animal Care and were performed in strict accordance with IACUC guidelines.

### Histology

Five µm sections of formalin fixed, paraffin-embedded mouse tongue tissue or tumors were stained with hematoxylin and eosin. Slides were examined with brightfield microscopy using the microscope and imaging software described for immunofluorescence analysis.

**Figure 5:**
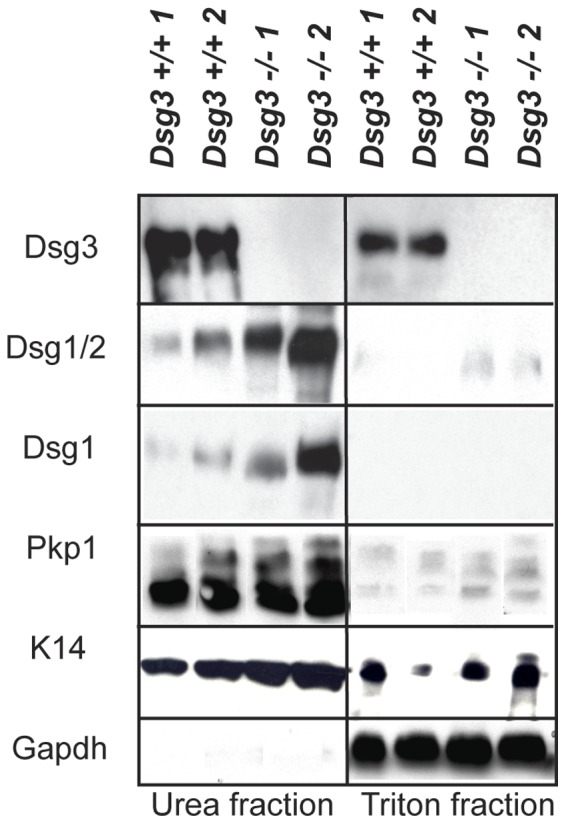
Dsg3 loss in the skin results in Dsg1 and Dsg2 upregulation. Western blot analysis showing both the Triton X-100-soluble and urea-only soluble fractions of mouse skin lysates from *Dsg3+/+* and *Dsg3−/−* mice. 1 and 2 denote samples from two mice. GAPDH serves as a loading control for the Triton X-100-soluble pool, while Keratin 14 serves as the loading control for the urea fraction.

### Tumor study

*Dsg3+/−* mice were intercrossed to obtain cohorts *of Dsg3+/+ and Dsg3−/−* mice on a 129/Sv;C57BL/6 mixed background. Specifically, 6 pairs of *Dsg3+/−* mice, of genetically similar background (all siblings or first cousins) were bred, and cohorts of 23 wild-type mice and 11 *Dsg3*−/− mice were generated for the tumor study. At 10 weeks of age, mice were exposed to chronic UVB light treatment (2.5 kJ/m^2^, three times a week, for 30 weeks). Mice were shaved on a weekly basis and treated using Kodacel-filtered FS40 sunlamps. Mice were placed 5 in a cage and allowed to roam freely during treatment. Cages were rotated along the shelf below the light bulbs before each treatment to compensate for uneven distribution of energy along the bulbs. Mice were monitored for tumor development by visual inspection.

### *In vivo* Apoptosis Assays

Cohorts of 10-week old *Dsg3* ***−*** */* ***−*** and *Dsg3* ***+*** */* ***+*** mice were generated. After shaving the dorsal skin of mice, they were placed underneath a Kodacel filter and allowed to roam freely in their cage during UVB treatment. Half of the dorsal skin was exposed to a one-time dose of 2.5 kJ/m^2^ of UVB irradiation while the other half was blocked with tape. 24 hours later, the dorsal skin of the mice was collected, processed, and immunostained for cleaved Caspase 3. Apoptosis, indicated by cleaved Caspase 3-positivity, was quantified in at least 2–3 cm of skin per mouse.

### Toluidine Blue Staining

Tissue samples were fixed overnight in 10% formalin, processed, and embedded using standard procedures. Samples were deparaffinized, rehydrated, and unmasked using Trilogy (Cell Marque) in a pressure cooker for 15 minutes according to the manufacturer’s instructions. Samples were then rinsed in phosphate buffered saline (PBS) and stained using toluidine blue [29]. Samples were mounted with Mowiol (EMD Chemicab).

## Results

### Dsg3 Loss Compromises Adhesion in Keratinocytes

To characterize the role of Dsg3 in cultured primary keratinocytes, we first analyzed the impact of Dsg3 loss on desmosome function using mouse keratinocytes grown in high calcium medium to induce desmosome formation. We first assessed the consequences of Dsg3 deficiency on the localization of individual desmosomal constituents using immunofluorescence analysis for various desmosomal components in *Dsg3+/+, Dsg3+/−,* and *Dsg3−/−* mouse keratinocytes (Fig. 1A). All desmosomal components examined displayed apparently normal membrane localization in *Dsg3*−/− mouse keratinocytes, similar to that observed in heterozygous and wild-type counterparts, indicating that *Dsg3* deficiency does not dramatically affect membrane targeting of other desmosome components. To ascertain whether desmosomes were functionally impaired, we used a solubility assay based on the fact that correctly formed desmosomal complexes can be solubilized only by chaotropic agents such as urea, while improperly assembled desmosomal components can be solubilized by the nonionic detergent Triton X-100 [31]. We assessed whether desmosomal components exhibited increased Triton X-100-solubility in the absence of Dsg3. Surprisingly, no differences were found in the solubilization properties of desmosome proteins in *Dsg3*−/− and wild-type keratinocytes, although increased levels of the other desmogleins, Perp, and Pkp3 were detected in the insoluble fraction of *Dsg3−/−* keratinocytes compared to controls (Fig. 1B). The ultimate test of an effect on cell-cell adhesion however, is an assay known as the mechanical dissociation assay. To determine whether Dsg3 loss compromises cell-cell adhesion in cultured keratinocytes, we subjected wild-type and *Dsg3−/−* keratinocyte monolayers to a mechanical stress and quantified the number of resulting fragments. We found that the number of fragments released was significantly greater in *Dsg3−/−* keratinocytes than in controls (Fig. 1C), revealing a defect in cell-cell adhesion in the absence of Dsg3, consistent with the *in vivo* adhesion deficits associated with Dsg3 loss [32], [33]. Together, these findings indicate that *Dsg3−/−* keratinocytes display defective desmosomal adhesion, although associated with only subtle changes in desmosomal components in the immunofluorescence and solubility assays we utilized.

### Dsg3 Deficiency Impedes Allograft Tumor Growth

Given that Dsg3 loss compromised cell-cell adhesion in keratinocytes, we sought initially to examine the consequence of Dsg3 loss to tumorigenesis using a keratinocyte-based allograft assay. We used *Dsg3−/−* and *Dsg3+/−* control keratinocytes transformed through activated H-Ras expression and *p53* inactivation because these represent key lesions for the development of squamous cell carcinoma (SCC) *in vivo* (Fig. 2A, B) [34], [35], [36]. We tested the tumorigenic potential of these cells by injecting them into the flanks of immunocompromised *Scid* mice and assessing tumor growth. After five weeks, at which time mice were sacrificed and tumor volume was calculated (Fig. 2C), all mice injected with transformed *Dsg3+/−* keratinocytes developed tumors, whereas only 80% of the mice injected with transformed *Dsg3−/−* keratinocytes developed tumors (data not shown). While tumor histology was similar in the two cohorts, the tumors derived from transformed *Dsg3−/−* keratinocytes showed a clearly reduced volume compared to tumors derived from transformed *Dsg3+/−* keratinocytes (Fig. 2C and D). These findings suggest that Dsg3 facilitates, rather than inhibits, tumor growth in transformed keratinocytes.

### Dsg3 Deficiency does not Promote UVB-induced SCC Development

The data from the allograft assays suggest that Dsg3 enables tumor growth *in vivo.* In contrast, we recently found that loss of another key desmosome component, Perp, promotes carcinogenesis in an autochthonous model for SCC development in the skin driven by UVB-exposure [29]. To investigate whether Dsg3 deficiency can, similarly to Perp loss, sometimes promote cancer, we sought to examine tumor development in *Dsg3−/−* mice. As reported, we noted *Dsg3−/−* mice display clear desmosomal defects in their stratified epithelia, including blistering in the oral mucosa and hair loss (Fig. 3A, B) [32], [33]. We thus tested whether desmosome compromise associated with Dsg3 deficiency could promote UVB-induced tumor development in the skin. We subjected 10-week old *Dsg3*−/− or control wild-type mice to chronic UVB treatment for 30 weeks (Fig. 3C). Tumor latency, size, and multiplicity were monitored throughout the study. Surprisingly, Kaplan-Meier analysis revealed no significant differences in tumor latency between the two cohorts (Fig. 3D). Similarly, the average tumor size was not clearly different between *Dsg3* null and wild-type mice (Fig. 3E). Histological analysis demonstrated that tumors arising in both wild-type and *Dsg3*−/− mice were SCCs and sarcomas, as described [37], and that these tumor types arose at similar proportions, irrespective of genotype (Fig. 3F). In addition, because our previous findings indicated that Perp deficiency not only stimulated tumor initiation but also SCC progression, we analyzed the tumor grade of SCCs resulting from chronic UVB exposure. Grading of tumors in both cohorts failed to reveal differences in the degree of SCC differentiation between the two cohorts of mice, with 50%, 30% and 20% of the SCCs in each cohort appearing well differentiated, moderately differentiated, and poorly differentiated, respectively (Fig. 3G). Collectively, these results indicate that Dsg3 loss has a minimal effect on UVB-induced SCC development. Thus, unlike Perp loss, Dsg3 inactivation is not sufficient to promote carcinogenesis in the epidermis upon UVB exposure.

### Apoptosis and Immune Cell Infiltration Appear Uncompromised by Dsg3 Loss

The dramatic enhancement of UVB-induced skin carcinogenesis in Perp-deficient mice is likely at least in part due to the fact that Perp loss compromises UVB-induced apoptosis, enhancing survival of cells that have sustained DNA damage and which may have accrued oncogenic mutations. Indeed, not only Perp, but also another desmosomal component, Dsg1, is critical for UV-induced apoptosis [29], [38]. To better understand the failure of Dsg3 loss to enhance UVB-induced skin cancer development, we therefore examined whether Dsg3 deficiency impaired the apoptotic response in the epidermis after UVB treatment. *Dsg3+/+* and *Dsg3*−/− mice were treated with 2.5 kJ/m^2^ UVB, and 24 hours later, the skin was analyzed for apoptosis using immunohistochemistry for cleaved caspase 3, a marker of apoptosis (Fig. 4A, 4B). Quantification failed to reveal, however, a difference in apoptotic indices in the epidermis of UVB-treated *Dsg3* null and wild-type mice, suggesting a rationale for the lack of enhanced tumor phenotype in UVB-treated *Dsg3−/−* mice.

Another mechanism through which Perp loss may contribute to UVB-induced skin cancer is through the recruitment of inflammatory cells that could promote cancer. Perp inactivation, combined with chronic UVB treatment for 19 weeks, led to an increase in immune cell infiltration, specifically mast cells, in the skin relative to wild-type controls [29]. We therefore compared mast cell infiltration in wild-type and *Dsg3−/−* mice after chronic UVB treatment by staining the epidermis with toluidine blue, an indicator of mast cells. Unlike the Perp-deficient mice, no significant differences were found in the number of mast cells in the skin of *Dsg3−/−* and control mice (Fig. 4C, D). Thus, despite manifesting clear deficits in cell-cell adhesion, no effects on UVB-induced apoptosis or immune cell recruitment were evident in the UVB-treated *Dsg3−/−* mice, providing a potential explanation for the absence of an increased tumor predisposition in *Dsg3−/−* mice in the UVB-induced carcinogenesis model.

### Desmosomal Cadherins are Upregulated in the Skin of *Dsg3*−/− Mice

Dsg3 is part of a family of desmosomal cadherins family comprising Dsgs 1–4 [39], and therefore other desmogleins could potentially at least partially compensate for Dsg3 deficiency. To understand the minimal effect of Dsg3 loss on UVB-induced SCC development, we sought to determine the effects of Dsg3 loss on desmosomal components in the skin using a solubility assay. While no clear difference in solubility pattern was noted between wild-type and *Dsg3−/−* skin, we did detect increased Dsg1 and Dsg2 levels in the *Dsg3−/−* skin, suggesting that these proteins are upregulated upon Dsg3 deficiency (Fig. 5). The upregulation of Dsg1 and 2 upon Dsg3 loss may compensate for deficiency in some Dsg3 functions and could explain the lack of phenotypes in the UVB-induced tumor model.

## Discussion

Although the role of adherens junctions constituents, particularly E-cadherin, in cancer development has been extensively studied, there has been limited functional investigation of the role of desmosomal cadherins in carcinogenesis. In this study, we set out to address the contribution of desmosomal cadherins to cancer development using a mouse genetic approach. In particular, we focused on Dsg3 and its role in SCC development, using two different models, and we found that the role of Dsg3 in cancer varies slightly according to context. *In vitro*, *Dsg3* null keratinocytes have a clear defect in cell-cell adhesion compared to wild-type cells, and interestingly, transformed *Dsg3−/−* keratinocytes form smaller tumors than transformed control keratinocytes in allograft tumor assays. These findings suggest that intact desmosomal adhesion facilitates tumorigenesis in this context. In contrast, using an autochthonous model for SCC development in the skin driven by UVB-exposure, we failed to detect any effect of altering Dsg3 status on tumor development. Moreover, unlike mice lacking the desmosomal component Perp, which are prone to UVB-induced SCC, no impairment in the apoptotic response to UVB and no increase in immune cell infiltration were observed in *Dsg3−/−* mice relative to wild-type mice. This observation thus suggests that in this model of cancer, Dsg3 loss does not promote or inhibit tumorigenesis.

Our observation that Dsg3 deficiency in transformed mouse keratinocytes impairs allograft tumor growth suggests that, in certain settings, Dsg3 may have oncogenic activity. We showed that in mouse keratinocytes, Dsg3 deficiency induces a defect in cell-cell adhesion, and therefore compromised adhesion in keratinocytes could account for the failure of tumors to form efficiently in the absence of Dsg3. This result is in keeping with the observation that Dsg3 knockdown can impede the growth of human head and neck cancer cell lines in xenograft assays [26]. Moreover, this observation is reminiscent of the inhibition of tumorigenesis we observed previously in Perp-deficient mice subjected to the DMBA-TPA two-step skin carcinogenesis protocol [40]. It may be that lack of cell-cell adhesion impedes survival of incipient tumor cells, thereby limiting tumor development in such instances.

Unexpectedly, there was no enhanced tumor development in *Dsg3−/−* mice relative to controls after chronic UVB treatment, in contrast to our previous observation that Perp deficiency in the epidermis promotes SCC development. The lack of increased SCC development in the *Dsg3−/−* mice may relate to the fact that Dsg3 is part of a multiprotein family and that other family members compensate for its loss. Redundancy or compensation by other family members is well-precedented, such as with the Retinoblastoma family of tumor suppressors, where multiple family members must be inactivated for mice to develop retinoblastomas [41]. Consistent with this notion, we observed that Dsg3 loss in the skin results in the upregulation of other members of the desmoglein family, particularly Dsg1. The notion that Dsg1 can compensate for Dsg3 is supported by experiments in which transgenic expression of Dsg1 in the telogen club, under the control of the K14 promoter, in *Dsg3−/−* mice rescues the balding phenotype characteristic of these mice [42]. Therefore, a possible explanation for the difference in cancer predisposition of mice deficient for Perp and for Dsg3 following chronic UVB treatment is that the potential consequences of Dsg3 deficiency may be compensated by induction of the other desmogleins. It would be very interesting in the future to test this notion by examining UVB-induced SCC cancer in mice deficient for multiple desmogleins to determine if compound mutants display enhanced skin cancer development. In addition, it is notable that in another model, combined oncogenic K-Ras expression and *Dsc3* deletion in mouse skin accelerated skin cancer development relative to Dsc3-expressing controls, suggesting a tumor suppressor role for Dsc3 [43]. As Dsc3 and Dsg3 have similar pattern of expression in the epidermis and are thought to be partners, it may be that Dsg3 loss promotes tumorigenesis in some other context, such as upon activated Ras signaling.

Alternatively, another explanation for the lack of tumor predisposition in *Dsg3−/−* mice is that desmogleins function differently from Perp and do not play roles as tumor suppressors. The lack of tumor suppressor activity of Dsg3 we observed is consistent with certain previous reports about the role of DSG3 in human carcinogenesis. For example, it has been shown that DSG3 is overexpressed in SCCs of the head, neck and lung, suggesting that it may act as an oncogene [26], [27]. Interestingly, DSG3 deficiency is linked to the blistering disease Pemphigus Vulgaris, in which autoantibodies directed against DSG3 in the serum of patients attack the protein and compromise desmosomal function, leading to severe blistering of the oral mucosa. This phenotype is clearly recapitulated in *Dsg3−/−* mice [32], [33]. However, the fact that a clear cancer predisposition has not been noted in these patients suggests further that loss of Dsg3 is not sufficient for cancer development in stratified epithelia. Understanding if and how desmogleins and Perp act differently are important areas for future investigation.

Desmosomes have been viewed for years as static structures required primarily for strong adhesion between keratinocytes. It has become increasingly clear, however, that the function of desmosomes is more complex. An increasing number of reports link desmosome function to proliferation, apoptosis, and migration, and therefore the role of desmosomal complexes in cancer development still remains to be better understood. Future analyses will better elaborate the roles of the various desmosomal constituents in carcinogenesis in different settings.
